# Supplementary material for: Impact of prior SARS-CoV-2 infection on perioperative cardiac, pulmonary and neurocognitive complications in older patients: Study protocol for an observative case control study
Source: PLoS One. 2025 May 12;20(5):e0323599. doi: 10.1371/journal.pone.0323599 (PMC12068606; doi:10.1371/journal.pone.0323599)
Supplement: S1 Fig — (PDF) [file pone.0323599.s001.pdf]

Supporting Figure S1

	STUDY PERIOD						
	Baseline	Day of surgery	Postoperative Day				
**TIMEPOINT**	Pre-surgery	0	*1*	*2*	*3*	*4*	*5*
**ENROLMENT:**							
In- and exclusion criteria, consent	x						
Demographic data	x						
Physical status (CCI, iADL)	x						
Main diagnosis, Type of surgery	x						
Medication	x						
Frailty (CFS and Fried criteria)	x						
MOCA	x						
COVID-Status	x						
**INTERVENTIONS:**							
Surgery procedure		x					
Laboratory parameters	x	x					
Anesthesia data		x					
**ASSESSMENTS:**							
Cardial complications		x	x	x	x	x	x
Pulmonal complications		x	x	x	x	x	x
Delirium (4AT und 3DCAM)		x	x	x	x	x	x
Other complications (PONV)		x	x	x	x	x	x
Patients' questionnaire (pain)		x	x	x	x	x	x
Extraction of clinical data	x	x	x	x	x	x	x
Use of interim reports		x	x	x	x	x	x
X-Ray, MRI, CT-Reports		x	x	x	x	x	x

* *CCI* Charlson Comorbidity Index, *iADL* instrumental activities of daily living, *CFS* clinical frailty scale, SF-36 short-form assessment of *MOCA* Montreal Cognitive Assessment, *4AT* 4A´s Test for delirium and cognitive decline, *3DCAM* 3-dimensional Confusion Assessment Method, *PONV* Postoperative Nausea und Vomiting
